# Enhancing Joint Attention Skills in Children on the Autism Spectrum through an Augmented Reality Technology-Mediated Intervention

**DOI:** 10.3390/children9020258

**Published:** 2022-02-14

**Authors:** Patricia Pérez-Fuster, Gerardo Herrera, Lila Kossyvaki, Antonio Ferrer

**Affiliations:** 1 Department of Educational Psychology and Psychobiology, School of Education, Universidad Internacional de La Rioja, 26006 Logroño, Spain; 2 Autism and Technologies Laboratory, University Research Institute on Robotics and Information and Communication Technologies (IRTIC), Universitat de València, 46010 Valencia, Spain; Gerardo.Herrera@uv.es; 3 Department of Disability, Inclusion and Special Needs (DISN), School of Education, University of Birmingham, Birmingham B15 2TT, UK; a.kossyvaki@bham.ac.uk; 4 Department of Developmental and Educational Psychology, Faculty of Psychology, Universitat de València, 46010 Valencia, Spain; antonio.ferrer@uv.es

**Keywords:** autism, joint attention, intervention, augmented reality, technology, school, children

## Abstract

In the present study, the effects of an intervention based on an augmented reality technology called Pictogram Room were examined. The objective of the intervention was to improve the responding to joint attention (RJA) skills of gaze following and pointing in six children on the autism spectrum between 3 and 8 years old. A multiple baseline single-subject experimental design was conducted for 12 weeks in a school setting. Results indicated that all of the participant children improved performance in RJA following the intervention. Improvements were maintained over time and generalised to real-world situations. These findings demonstrate that autistic children can improve their RJA skills with a targeted and engaging intervention based on an accessible augmented reality technology tool.

## 1. Introduction

Joint attention (JA) can be defined as the shared focus occurring when one individual gets the attention of another towards an object or event by means of eye-gazing, pointing or other verbal or non-verbal indications. Two main areas of JA which have been widely researched are (a) initiating JA (IJA) and (b) responding to JA (RJA) [1,2,3,4]. IJA refers to the use of eye contact, gaze shifting and gestures to direct the attention of a social partner to a referent of interest [5,6] while RJA refers to response by gaze following, pointing or showing to enhance social interaction with others [7,8,9]. Particularly, gaze following has been considered an important RJA skill because it contributes to understanding what another person is thinking, feeling and intending to do [10,11,12] facilitating, therefore, the development of a Theory of Mind [13,14].

### 1.1. Differences in JA between Non-Autistic and Autistic Children

Children typically develop the ability to participate in JA interactions (e.g., coordinated eye gaze) at between six and twelve months of age [3,8,15,16] and they successfully gaze follow and point before 24 months of age [17,18]. However, JA skills have been identified as profoundly difficult among autistic children [19]. This difficulty can be present at the first stages of development but it also often remains throughout their lifespan. Research has indicated that IJA and RJA are two distinct forms of JA that develop differently—and are associated with different brain patterns [20]—as individuals grow older [21,22,23]. According to these studies, when children are at the preverbal stage, significant differences can be observed in the RJA and IJA skills of autistic children compared to non-autistic children, the first population being affected, especially in the ability for RJA. More precisely, it has been found that autistic children engage less than non-autistic children in situations in which the skills of gaze following, showing and pointing are necessary [24,25,26]. However, that difficulty with RJA in autistic children becomes less evident as their language or mental age exceed 30 months of typical development. IJA differences between non-autistic and autistic children, instead, do not appear to decrease at 30 months of age but continue throughout the preschool period and, in some cases, they are still evident in adulthood [9,19,27,28]. Therefore, RJA difficulties are critical to many aspects of development in autistic children, with early language development being the most prominent one [27,29], whereas IJA difficulties appear to be more associated with developmentally chronic differences associated with autism symptoms [30].

### 1.2. Intervention Studies for Enhancing JA Skills in Autistic Children

Literature to date suggests that a wide range of intervention methods have been effective in increasing IJA and RJA skills, partially contributing to the improvement of other critical developmental areas, including social and language skills such as social initiations, positive affect, imitation and spontaneous speech in autistic children [31,32]. For example, Applied Behaviour Analysis (ABA) techniques such as prompting, modelling, reinforcement and different combinations of these three, have been effectively delivered by therapists [33,34,35,36] and peers [37]. Pivotal Response Trial (PRT) intervention methods—delivered by therapists [38,39], parents [40,41,42], siblings [43] and peers [44]—and interventions based on Reciprocal Imitation Training (RIT) [45,46] have also been found to be effective. The Early Start Denver Model (ESDM) [47] has been reported to be effective for increasing IJA and RJA skills in autistic children between 12 months and 5 years of age [48,49,50]. Furthermore, intervention methods that have been specifically developed for the enhancement of JA have been found to be effective. On one hand, Joint Attention-Mediated Learning (JAML) provides methods to principally increase parental responsiveness to their children’s IJA and RJA bids. Two JAML studies reported significant improvements in RJA [51,52]. On the other hand, Joint Attention, Symbolic Play, Engagement and Regulation (JASPER) is a theory- and evidence-based targeted approach to early developmental-behavioural intervention for autistic children [53]. JASPER has been delivered by therapists [54,55,56], teachers [57] and parents [58,59,60] leading to improvements in JA skills, and other principal related areas such as language development [61,62,63]. Limitations of the aforementioned studies can be summarised as follows: (a) the interventions in question were either too long or intense making them incompatible with many individuals’ acknowledged time constraints, (b) the therapists, teachers and carers had to invest much money, time and effort in mastering the intervention before delivering it, and (c) generalisation was scarcely reported.

### 1.3. Technology-Mediated Intervention Studies for Enhancing JA Skills in Autistic Children

The fast development of innovative technology and the affinity that many autistic people tend to display for it have contributed to a rise in Technology-Mediated Interventions (TMI) studies, some of which have focused on the enhancement of JA skills. The Virtual Reality (VR)-based Joint Attention Skills Learning (JASL) system [64] and the robots Troy [65], Kaspar [66] and Nao [67,68] have been used with autistic children between 2 and 12 years old in a variety of settings ranging from laboratories to schools. Results showed that the above-mentioned TMI were effective for the enhancement of IJA and, especially, RJA skills in 21 of the total 23 participants included in these studies. However, several limitations are likely to prevent replication of these studies; the lack of availability in the market of the hardware (HW) and/or software (SW) technology used and the necessity to involve the developer to operate the HW and/or SW during the intervention sessions are the two most noteworthy ones. A recent systematic review on the use of the above-mentioned robots among other social robots for JA development in autistic children [69] indicates that although children tend to show positive reactions to robots as social partners, the clinical relevance of the improvements and the generalisation of the skills have not been sufficiently demonstrated yet.

### 1.4. Augmented Reality

**Figure 1:**
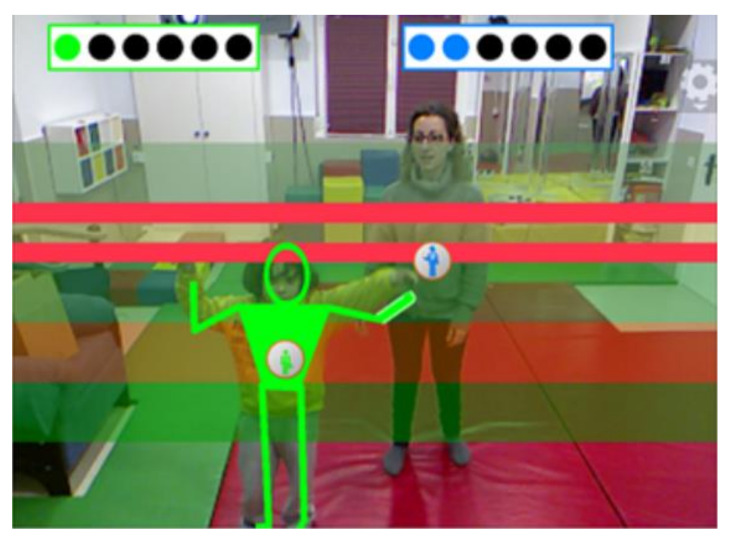
A child playing Pictogram Room with the support of his teacher. *Note.* This image does not belong to our study, it is just a sample adopted from the Pictogram Room’s website: http://www.pictogramas.org/proom (accessed on 13 January 2022).

Augmented Reality (AR) has been defined as a technology that combines the information one perceives from the real world with information generated by a computer in real-time [70]. Compared to other technologies, AR’s main advantages for autistic individuals are the following: (a) it can be considered a tangible presence [71] since the process of perceiving and acting in the world is much more natural than in VR where the reality, although it can be potentially authentic [72], is simulated; (b) it includes body representation which can help autistic individuals to perceive themselves and track their own movements contributing significantly to maintaining body awareness [73]; and (c) since AR combines both real and virtual characteristics, it can be a useful tool for scaffolding the generalisation of skills learned in a virtual world to the real world [74]. Previous studies have highlighted the positive impact that AR, used in combination with other technologies including smartphones, smartglasses, mirror-based systems and Kinect (i.e., a depth sensor that captures the body postures and movement of several individuals through infrared cameras in real-time), can have on the development of attention, social communication and social interaction skills in autistic individuals [75,76,77,78,79]. A systematic review that included some of these studies, among others, concluded that the use of AR produces positive results in autistic people, especially in children [80]. A free to download commercial software that has been specifically developed for autistic individuals is the Pictogram Room (see Figure 1; http://www.pictogramas.org/proom, accessed on 13 January 2022). The Pictogram Room is a Kinect-based AR system that has been available since 2011 and includes video games that have been designed for enhancing a wide variety of skills, including JA [81,82]. Although its effects on JA have not been evaluated, this AR system has been found to be a useful tool to evaluate body knowledge [83] and was effective at enhancing sensory-motor skills in five autistic children between 4 and 6 years old in a school setting [84].

In light of up-to-date literature, and based on the potential of AR and, specifically, the Pictogram Room, it was hypothesised that a short intervention delivered by a teacher in a school setting, using this tool, with a targeted game and personalised stimuli would improve RJA skills in autistic children.

### 1.5. Purpose of This Study

The current study aimed to explore the impact of an AR-TMI, using the Pictogram Room tool, on the RJA skills of gaze following and pointing in autistic children. To ensure the rigour and strength of the research report, the quality guidelines for evaluating and determining evidence-based practices in autism [85] were followed throughout the study.

## 2. Materials and Methods

### 2.1. Participants

Six autistic children without visual difficulties of preschool and primary school age were recruited from a mainstream public school located in the province of Valencia (Spain) to participate in the study. Further information on the participants’ characteristics is shown in Table 1.

All six children attended classes in an autism unit within the mainstream school that was formed of (a) one teacher specialising in speech and hearing difficulties who focused on children’s communication, language and speech; (b) one special education teacher whose responsibility was mainly teaching and assessing the curriculum goals; and (c) two learning support assistants who primarily taught children independence and self-help skills such as hygiene, eating and transitioning. Only the two teachers of the unit participated in the study. The teacher specialising in speech and hearing difficulties (hereafter referred to as “Teacher A”) was a 28 year old female who had a B.Sc. in Special Education with a major in speech and hearing difficulties, a B.Sc. in Speech Therapy and six years of experience in teaching autistic children. The special education teacher (hereafter referred to as “Teacher B”) was a 41 year old female who had a B.Sc. in Special Education and 18 years of experience with autistic children. Teacher A (a) taught the children how to play with the Pictogram Room, (b) was their play partner at the sessions and (c) participated in a number of assessments that were administered to the children. Moreover, Teachers A and B helped with the organisation of each child’s timetable and provided valuable information regarding the children’s special educational needs, skills and preferences.

The autism unit layout and the organisation and administration of activities were based on the Treatment and Education of Autistic and related Communication Handicapped Children (TEACCH^®^) program [88]. One of the children (i.e., P3) used verbal language to communicate whereas the other five children used the Picture Exchange Communication System (PECS) [89] with symbols and books to communicate and follow their daily routines and activities.

### 2.2. Setting and Technology Equipment

**Figure 2:**
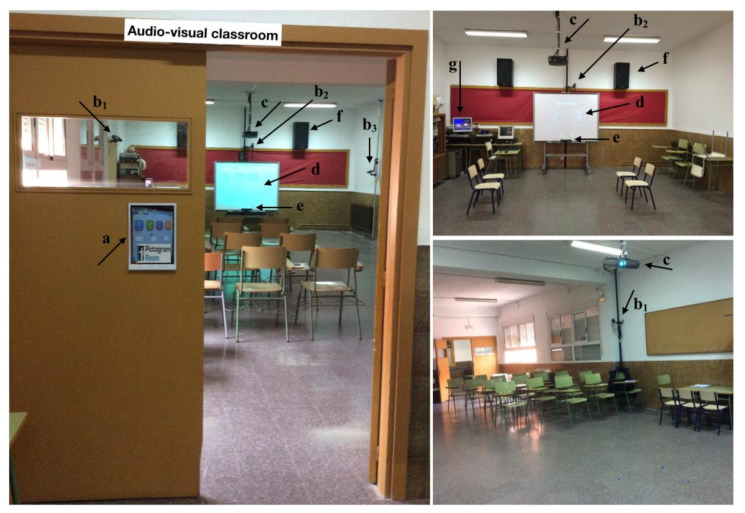
Room arrangement: (**a**) board for the children to place the PECS symbols, (**b_1,2,3_**) video cameras, (**c**) projector, (**d**) IDW, (**e**) Kinect, (**f**) speakers, and (**g**) PC screen displaying the video captures. *Note*. Four chairs were placed in front of the IDW to show the dimensions of the play area but they were not used during playtime. In the study sessions, the PC screen (i.e., (**g**)) was hidden behind a paper screen so children could not see it and get distracted.

The room where all assessment and intervention sessions were conducted was located in the school and was called the audio-visual classroom (see Figure 2). Its frontal area was spacious enough (i.e., more than 10 × 10 feet) for interacting with the Pictogram Room, it had tables and chairs for the children and good light conditions (i.e., natural light that could be controlled with blinds). With regard to technology, the room was originally equipped with one Epson EMP-82 LCD Projector and one Interactive Digital Whiteboard (IDW) (i.e., SMARTBoard™). To complete what was needed for the use of the Pictogram Room, one Personal Computer (PC) with Windows Operative System (OS), and one Kinect for Xbox were added. Additionally, another PC with Windows OS, one AKG UHF 40 pocket transmitter and stationary receiver, and three Samsung SCC-301P video cameras, statically located in different points of the room, were used for video-recording the sessions. See the final look of the room in Figure 2.

### 2.3. The Pictogram Room

**Figure 3:**
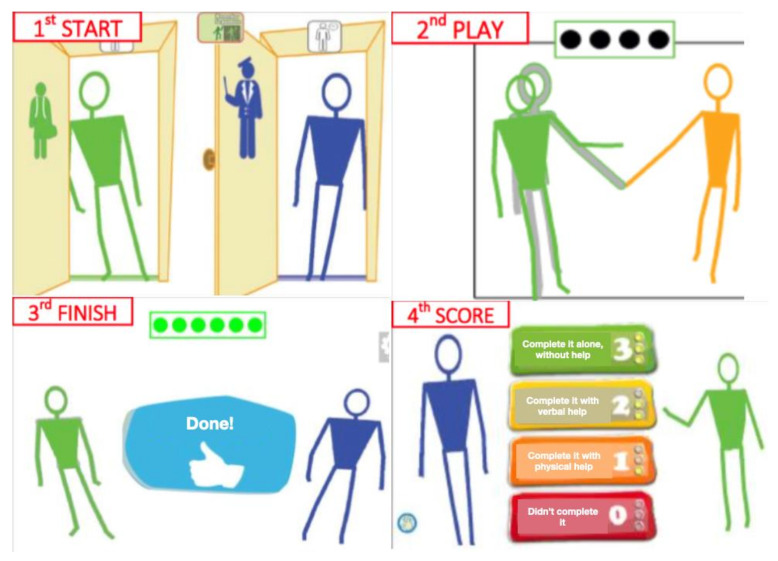
The Pictogram Room’s sequence of play. Adopted from the pedagogical *Guide for parents and tutors* that is available at http://www.pictogramas.org/proom (accessed on 13 January 2022).

The Pictogram Room consists of four sets of video games: (a) The body, (b) Positions, (c) Pointing and (d) Imitation. Each set contains multiple games that are ordered by difficulty and were developed to train different skills such as body knowledge, imitation and JA. There are 80 games in total. Most of them can be played either by only one student or collaboratively with the teacher. Figure 3 shows the Pictogram Room’s sequence of play.

When the student plays collaboratively, each game starts by asking both players to stay still in a natural posture for three seconds in front of two virtual doors (one for each player) at a specific distance from the screen (i.e., 8 feet) to allow the Kinect to calibrate their bodies. When the student plays individually, only one door is displayed and 6 feet of distance between the player and the Kinect are required. Players should keep that initial distance so that Kinect can adequately track them and ensure accurate performance during playtime. The length of each game depends on the number of goals the player has to achieve—this is indicated with black circles on the top of the screen which become green when a goal is achieved—and the time it takes to achieve these goals. Once the player completes each game, a reward is displayed on the screen and a scoring system appears to allow the teacher to give a score to the student. The scores are as follows: 0 points, if s/he does not complete it; 1 point, if s/he completes it with physical help; 2 points, if s/he completes it with verbal help; and 3 points, if s/he completes it without help. This data can be recorded for later analyses. All games can be adapted to each student’s learning pace and they can also be personalised according to their visual (i.e., images and videos) and musical (e.g., melodies and songs) preferences through the Pictogram Room website, which automatically synchronises with the computer program.

### 2.4. Materials

Four assessment tools were administered to the children by the first author (hereafter referred to as “the researcher”) for describing participant characteristics and for evaluating RJA skills (see Table 2).

**Figure 4:**
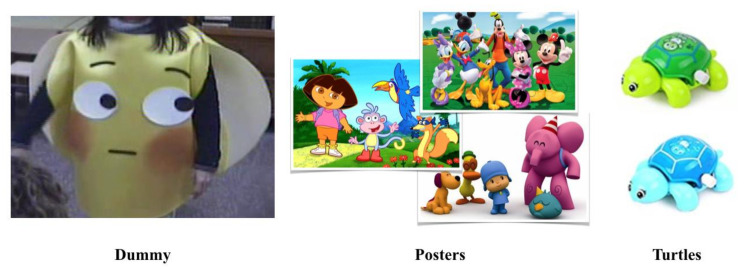
Items used for developing ad-hoc RJA skills assessments.

Besides, three types of items were used for developing ad-hoc RJA skills assessments: a dummy, posters and turtles (see Figure 4).

The Dummy consisted of a felt-based handcrafted face with an emotionally neutral expression and two fixed white circles simulating the eyes, with two movable black circles simulating the irises. The two irises were attached to the eyes with Velcro. This allowed the evaluator to move them around the eye area to show different points of gaze. The dummy had three holes (one for the head and two for the arms of the evaluator) to be worn as a costume. The Posters were a set of A4 laminated posters with colourful images of cartoon characters. The Turtles were two blue and two green 1.5 × 2.5 × 3.5 in. plastic turtles with a simple mechanism based on a small wheel to be rotated for making them move short distances. These items were used for developing four RJA skills assessments:

1. Which poster is s/he looking at? The researcher and the child sat opposite to each other and the teacher next to or behind the child. The researcher took two posters and positioned them next to her eyes, one on the left and one on the right. Then, she moved her eyes to look at one of them and Teacher A asked: “Which poster is she looking at?”. To obtain a correct response, the child had to point to or take hold of/take away the poster the researcher was looking at. The cartoon characters of the posters were familiar to the children but their favourite ones were not used to avoid impulsive choices based on their preferences. The assessment consisted of 10 trials in which the posters position and the gaze direction were randomly altered.
2. Which turtle is s/he looking at? The researcher and the child sat opposite each other and the teacher next to or behind the child. The researcher held two turtles next to her eyes, one on the left and one on the right. Then, she moved her eyes to look at one of them and Teacher A asked: “Which turtle is she looking at?”. To obtain a correct response, the child had to point to or take hold of/take away the turtle the researcher was looking at. The assessment consisted of 10 trials. On the first five trials, the turtles had the same colour (two blue or two green), and on the subsequent five trials they had different colours (one blue and one green). Two same-coloured turtles were used for ensuring that the children did not choose their favourite colour turtle but they selected the target they thought was the correct one.
3. Which poster is the dummy looking at? and 4. Which turtle is the dummy looking at? The same procedure as in (1) Which poster is s/he looking at? and (2) Which turtle is s/he looking at? assessments was followed but the researcher wore the dummy costume, the objects were placed next to the dummy’s eyes and she randomly alternated the dummy’s gaze direction. The teacher was the one to ask the child “Which poster is the dummy looking at?” or “Which turtle is the dummy looking at?”.

The questions addressed to the participants were formulated based on their level of receptive language, which was obtained by Teacher A prior to the study sessions, to guarantee their comprehension. Regardless of the response that the participants gave at each trial (i.e., correct or incorrect), they were given time (a few seconds) to interact with the object (i.e., touching the poster, making the turtle move) in between each trial.

### 2.5. Research Design

A multiple baseline single-subject experimental design (SSED) [94] across three groups of two participants was conducted. This research design was implemented to control the participants’ developmental maturation and exposure to the researcher and the intervention setting. This also allowed for the assessment of several target behaviours simultaneously to test the effectiveness of an intervention. In a multiple baseline SSED, intervention trends can be easily identified for each child and within-subject variability can be measured, which is highly desired when working with individuals with special educational needs due to their heterogeneity. As recently highlighted, these designs can be used with any sample size and help researchers and practitioners to (a) bridge the research–practice gap, (b) use no more resources than needed, (c) respect the dynamic nature of learning and (d) appreciate functional diversity and respond to research questions accordingly [95].

### 2.6. Procedures

Before the study sessions started, the researcher went to the school on several days and spent a long time at the autism unit to get to know the teachers, learning support assistants and children and for them to get to know her. For the anticipation of each study session, two new PECS symbols were added to the children’s PECS repertoire: one symbol was the researcher’s photo (including her name) and another symbol was a picture representing the Pictogram Room. Each child was accompanied by Teacher A, Teacher B, one learning support assistant or the researcher from the autism unit to the audio-visual classroom and back to the unit. For each transition, the child held the PECS symbol(s) and placed it afterwards on the board of the unit or on the board located at the entrance door of the audio-visual classroom (see Figure 2). Children could carry their PECS communication books and had them available throughout each session so that they could communicate anytime if they wanted to. The researcher was present in all of the study sessions ensuring the fidelity of the implementation of the intervention and providing continuous technical support (e.g., reassigning the correct role to the student and the teacher when the Kinect failed at recognising correctly their roles as a consequence of the players exchanging positions), as well as educational aids (e.g., modelling the children to perform successfully whenever Teacher A requested her help).

The length of the study (i.e., eight weeks excluding the follow-up assessments) coincided with the number of consecutive weeks the children attended school without holidays that could interrupt the implementation of the intervention. The full study lasted 12 weeks and consisted of the following phases (see Table 3):

- Pre-baseline Phase (week 1)

One-off assessments. SCQ and Leiter-R were administered to participants by the researcher. The scores obtained in the SCQ confirmed participants’ autism diagnoses (DSM-5) and their levels of severity (GARS-2). The Leiter-R scores showed different levels of ID (i.e., borderline, mild and moderate) for the participant children (see Table 1). The researcher also went to the school to observe the children in the autism unit, their mainstream classrooms and playground. Besides, she informally interviewed Teachers A and B to get useful information (e.g., abilities, special needs, interests, preferences) of the children for the customisation of the Pictogram Room. As a result, personalised rewards were chosen for each child for the intervention sessions (e.g., songs and videos), the volume of the Pictogram Room games was turned down for P3 due to his auditory hypersensitivity, and a digital clock was added to an iPad to visually remind the children throughout each session how long they had left.

Pre-assessments. The ADOS-2 and ESCS tests and the Which poster is s/he looking at? and Which turtle is s/he looking at? assessments were administered to participants by the researcher. Since the outcomes of the four assessments indicated that all children had difficulties in RJA, all six participants were included in the study. Considering the length of the study, three baselines of different duration were conducted. Hence, participants were randomly assigned to three different groups: Group 1 was formed of P1 and P2, Group 2 included P3 and P4, and Group 3 consisted of P5 and P6. After this phase, the researcher visited the school three non-consecutive days a week for six weeks (i.e., from week 2 to week 7) for running one session with each participant a day resulting in three sessions per child per week.

- Baseline Phase (weeks 2–4)

The Baseline Phase had a different duration for each group: Group 1 completed three sessions (week 2), while Group 2 and Group 3, completed six (weeks 2–3) and nine sessions (weeks 2–4), respectively. This phase consisted of 15-min sessions for evaluating the children’s performance with the Which poster is the dummy looking at? and Which turtle is the dummy looking at? assessments.

- Learning Phase (weeks 3–5)

The purpose of this phase was to teach the participants how to play with the Pictogram Room. This consisted of three sessions for all groups. Group 1 went through the Learning Phase in week 3, while Group 2 and Group 3 went through it in weeks 4 and 5, respectively. This phase consisted of 30-min sessions that included two parts: (a) the familiarisation with the Pictogram Room (15 min) and (b) the Which poster is the dummy looking at? and Which turtle is the dummy looking at? assessments (15 min). The familiarisation part became the first encounter with the Pictogram Room and in order to facilitate the learning process, the children played a very motivating and relatively easy game called *Touch*, which belongs to *The body* set of video games and does not focus on JA skills. The children played individually while Teacher A was providing instruction and support. They saw their live images, as in a mirror, reflected on the screen. This game consisted of one static window (level 1), one moving window (level 2), one fast-moving window (level 3) and one moving window surrounded by other distracting stimuli (level 4). When the player touched the window, this opened and each child’s favourite video played for a few seconds. After six successful trials, which were indicated with green circles on the top of the screen, the level was mastered. Then, the researcher gave a score according to the player’s performance (i.e., 0–3). When level 4 was reached, the game was completed. Participants played as many level games as they could during each session and all of them entered level 4 by the third session.

- Intervention Phase (weeks 4–7)

**Figure 5:**
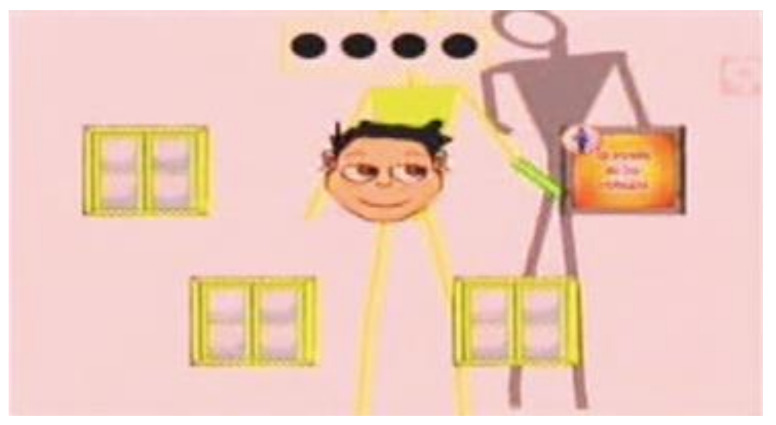
P2 playing level 4 of *Gaze following* game in the Pictogram Room with Teacher A during the Intervention Phase.

The intervention Phase consisted of six sessions for all groups. Group 1 went through the Intervention Phase in weeks 4–5, while Group 2 and Group 3 went through it in weeks 5–6 and 6–7, respectively. This phase consisted of 30-min sessions that included two parts: (a) the intervention with the Pictogram Room (15 min) and (b) the Which poster is the dummy looking at? and Which turtle is the dummy looking at? assessments (15 min) to measure changes in the children’s RJA skills straight after each engagement with the Pictogram Room. The intervention with the Pictogram Room consisted of the use of a game called *Gaze following*, which belongs to the *Pointing* set of video games focusing on JA skills. As in the learning phase, the children played individually while Teacher A was providing instruction and support. They saw their skeletons –instead of their live images– reflected on the screen (see Figure 5). The teacher was grey and the child was green.

This game consisted of a virtual child’s face with two big eyes that appeared surrounded by two (level 1) or four (levels 2–4) closed windows. The virtual child then pointed to and looked at (levels 1–2), or just looked at (levels 3–4) one of the windows. The teacher then asked the player: “Which window is the child looking at?”. If the player touched the window that the virtual child was looking at, the window opened and the child’s favourite video played for a while. If the player touched any other window, a specific sound was produced and all the windows were gone in order for another trial to start. A common quiet sound was chosen to signify the wrong answer so participants were not tempted to respond intentionally wrong to hear it. This was checked by observing that none of the participants touched the wrong window repeatedly across trials. After four successful trials, the level was mastered. Then, the researcher gave a score according to the player’s performance (i.e., 0–3). When level 4 was reached, the game was completed. Participants played as many level games as they could during each session and all of them entered level 4 by the fifth session.

- Post-intervention Phase

Post-assessments (week 8). As in Pre-assessments, the ADOS-2 and ESCS tests, and the Which poster is s/he looking at? and Which turtle is s/he looking at? assessments were administered by the researcher.

Follow-up assessments (week 12). One month after the end of the intervention, both the Which poster is s/he looking at? and Which turtle is s/he looking at? assessments were administered to all participants once on the same day and the Which poster is the dummy looking at? and Which turtle is the dummy looking at? assessments were administered to all participants twice, one per day, on two non-consecutive days.

To the researcher’s knowledge, participants never used the Pictogram Room (or any other AR system) before this study and they were not given the option to play with the Pictogram Room beyond the study sessions until this was over after week 12. Teachers A and B had previously made a few attempts to enhance children’s RJA with reinforcement-based methods (e.g., if the child followed the teachers’ gaze by pointing to the target object, the child would get a reward). However, they reported that they were no longer doing so as no improvements were observed because the method did not keep the children engaged. Thus, during the time the study was conducted, no other specific intervention program for enhancing JA skills was implemented at the school, and the same holds for the therapy centres where participants received different types of intervention (e.g., music and speech therapy).

Teachers A and B were informed about the general purpose of the study but they were blind to the research design applied. Also, Teacher A was asked not to reinforce children’s right answers and not to correct mistakes during assessments. Two independent blind raters (i.e., undergraduate education students) received training on the measured variables. Both coded all the assessment video recordings and Inter-Observer Agreement (IOA) and Cohen’s Kappa coefficients were calculated.

### 2.7. Data Preparation and Analysis

#### 2.7.1. Operationalisation of the Measured Variables

All measured variables are presented in Table 4.

The first variable (i.e., v1) refers to the participants’ performance within the Pictogram Room games. As explained in the procedures section, each game had four levels and one score was given to the participant after each level was achieved. Since each participant could achieve a number of levels, and some could even complete games more than once in the same session, each participant obtained many different scores in each session. The score that was given to v1 for each session was the lowest obtained by the participant across all the level games played in that session. This was to show the differences in the levels of support required by the children when they entered a new level game.

#### 2.7.2. Data Analysis

Scores obtained for v1 were visually analysed to observe performance improvement within the Pictogram Room games throughout the study. IOA coefficients were calculated for v2–v7 as the number of agreements between the two raters divided by the number of agreements plus the number of disagreements. Cohen’s Kappa was also calculated for v2–v7 by measuring the agreement between the two raters and subtracting agreement due to chance [96]. IOA can range from 0 to 1, with values higher than 0.80 indicating good IOA. Kappa can range from −1 to 1, and values higher than 0.70 indicate good inter-rater reliability. Besides, Percentage of All Non-Overlapping Data (PAND) [97] and Pearson Phi coefficients were calculated for v2–v3 using IBM SPSS Statistics 27.0 to assess the effectiveness of the intervention, given that these statistical techniques are suitable for identifying significant differences between scores obtained in baseline and intervention phases. Scores obtained for v4–v5 at pre, post and follow-up assessments and scores obtained for v6–v7 at pre- and post-assessments were compared to evaluate generalisation of the target skills. Additionally, Reichow et al.’s [85] single subject research quality indicators were applied to analyse the research report rigour and strength.

## 3. Results

### 3.1. Participant Improvement within the Pictogram Room Scoring System

**Figure 6:**
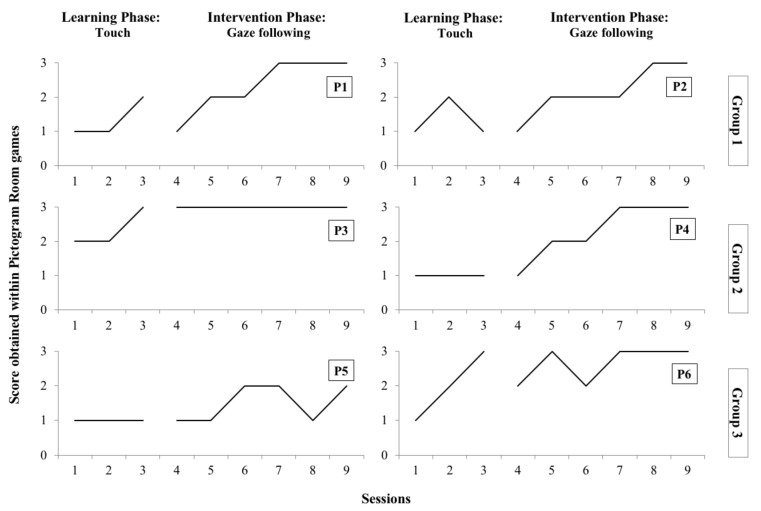
Participants’ scores within the Pictogram Room games.

Scores that were given to the participants within the Pictogram Room after they completed each game level showed that they all significantly improved their performance as the study progressed. The scores for each of the six participants are shown in Figure 6.

They were all engaged in the games from the first session and they completed all the levels of the games they played. However, they needed physical or verbal support to complete the games in the first couple of sessions. Particularly, P4 needed significant physical support at the beginning because instead of looking at the screen (i.e., AR environment), he was looking around to find the stimuli in the real world. However, in the second session of the Intervention Phase, he did not need any physical support and mastered playing the game independently in the last three sessions. Also, P4 and P5 required significant physical support in the first sessions because instead of keeping their initial position they tended to go to the IDW to touch the stimuli on it. Trend changes were observed in the third session of the Learning Phase for P2, and in the fifth and third sessions of the Intervention Phase for P5 and P6, respectively. These changes showed the impact that entering the most difficult level (i.e., level 4) of the *Touch* and *Gaze following* games had on these children’s performance. Towards the end of the Intervention Phase, all children except P5 performed all the levels of the games independently.

### 3.2. The Effectiveness of the Intervention in the Participants’ RJA Skills

**Figure 7:**
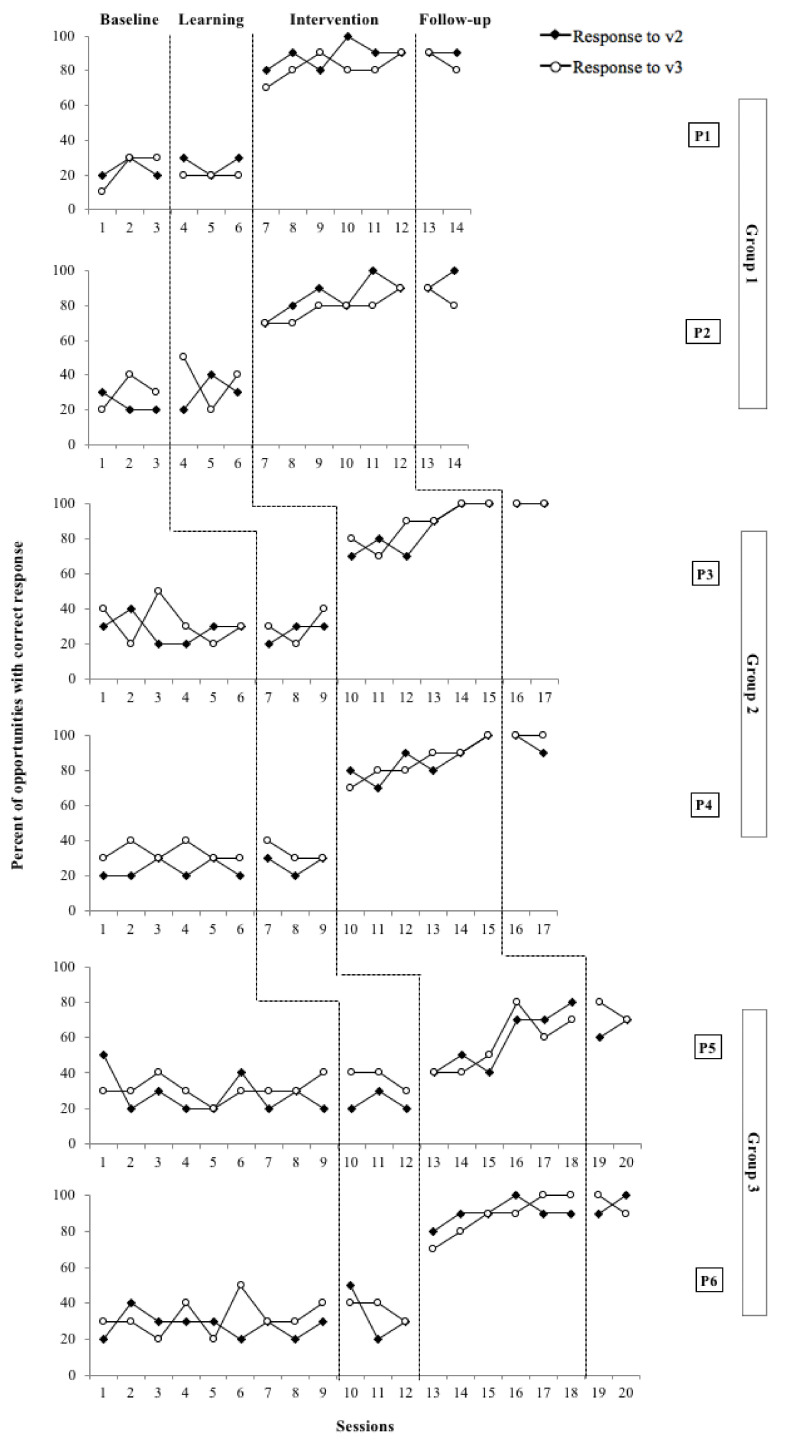
Participants’ performance in v2 (i.e., posters) and v3 (i.e., turtles) throughout the study.

The intervention was effective for all participants in terms of enhancing their RJA skills. At later stages of the Intervention Phase participants followed the dummy’s gaze and pointed to the object of shared attention (i.e., poster or turtle) between the dummy and the participant. IOA and Kappa coefficients for both v2 and v3 were 1, which shows perfect inter-rater reliability. Results have been plotted and are shown in Figure 7.

Participants responded in a very similar way to both v2 and v3 throughout the study. Therefore, participants did not perform differently when they had to gaze follow and point to one of the two objects when these were different (i.e., posters in v2) compared to when the two objects were the same (i.e., turtles in v3). Also, no significant differences were found when the children had to gaze follow and point to one of the two turtles when they had a different colour compared to when they had the same colour. No improvements were observed in any of the participants during the Baseline Phase or Learning Phase for any of the two variables. When the intervention with the Pictogram Room was introduced (Intervention Phase), remarkable improvements in RJA were observed. All participants except for P5 experienced significant improvements from the first session of the Intervention Phase and did not present any overlapping point between the Baseline and Learning Phases, and the Intervention Phase. However, P5 did not show clear improvements until the fourth intervention session, presenting a few overlapping points between phases. P1 and P2 mastered pointing to the right poster after four and five sessions, respectively, but none of them mastered pointing to the right turtle. P3 mastered pointing to the right poster and the right turtle after five sessions. P4 mastered both variables in the last intervention session. P6 was able to master pointing to the right poster and the right turtle after four and five sessions respectively. P5 mastered neither pointing to the poster nor the turtle. At the follow-up assessment, all six participants maintained the ability to gaze follow the dummy and point to the target object.

An overall PAND of 98% was obtained for v2 and an overall PAND of 96% was measured for v3. This shows that the intervention was highly effective (PAND > 90%) for enhancing the abilities of gaze following and pointing to the target object in six autistic children. Besides, Pearson Phi coefficient of 0.96 (*p* < 0.01) was obtained for v2 and 0.92 (*p* < 0.01) for v3. This indicates a strong positive association (Phi > 0.70) between the intervention and the learning outcomes.

### 3.3. Generalisation of the Participants’ RJA Skills

RJA improvements that were found in following the dummy’s gaze were generalised to a real gaze (see results for v4–5 in Table 5).

The comparison between pre- and post-assessments revealed that all participants improved at following the researcher’s gaze and pointing to the target posters and turtles. Follow-up assessments indicated that improvements were maintained a month later. The L/R RJA scores in ESCS obtained at pre- and post-assessments (see results for v6 in Table 5) also indicated improvements in all children’s RJA skills. The Behind RJA scores showed generalisation to correctly gaze follow and point to the target object when they were placed behind the child. Finally, RJA scores in ADOS-2 obtained at pre- and post-assessments (see results for v7 in Table 5) revealed that three of the six participants performed significantly better after the intervention: P2 and P6 followed the researcher’s pointing and they successfully looked at the target object, P3 used the orientation of the researcher’s eyes as a cue to look effectively at the target object. IOA and Kappa scores indicated perfect inter-rater reliability for v4–5 and good inter-rater reliability for v6–7 (see Table 5).

### 3.4. Research Report Rigour and Strength

The data obtained through Reichow et al.’s [85] evaluative method indicated that all primary quality indicators were rated high, and there was evidence for five of the six secondary quality indicators (see Table 6). According to the guidelines for the determination of research report strength ratings of this method, this study can claim to have produced a strong research report.

## 4. Discussion

### 4.1. Findings Summary

The results obtained in this study showed that the implemented AR-TMI was effective for all six participants. More precisely, the six children improved their abilities for following the gaze of a dummy and pointing to the object that the dummy was looking at. After the intervention, the six children demonstrated generalisation of the skills to follow the researcher’s gaze and point to the target object she was looking at. Additional pre- and post-assessments also showed generalisation of RJA skills to a novel situation in which the target object was placed behind them and the children had to turn their heads to look at it. Maintenance of the skills was observed one month after the end of the intervention.

### 4.2. Findings Supporting Previous Studies

Overall, this study showed that although the participant children had difficulties in gaze following and pointing prior to the intervention, they improved these skills. Such results have been evidenced not only within the Pictogram Room but also in all the scores obtained in the assessments that have been conducted without the use of technology (i.e., Which poster/turtle is the dummy looking at?, Which poster/turtle is s/he looking at?, the ESCS and the RJA item of the ADOS-2). These findings add to the literature arguing that JA constitutes a difficulty for many autistic individuals that can be effectively addressed with appropriate interventions [6]. Furthermore, the findings of the present study are in line with previous empirical studies which have reported that RJA skills in autistic children can be enhanced using ABA techniques such as prompting, modelling and reinforcement methods [34,35,37], PRT [39,41,43], RIT [45,46], the ESDM [50], and targeted JA interventions such as the JAML [51] or the JASPER [55,59,61].

### 4.3. Advantages of the Applied AR-TMI

Beyond its positive effects as stated in the Results section, the authors believe that the implemented AR-TMI has a number of advantages which are summarised below.

- Length

Its length is shorter than that of other interventions which are either very intense (i.e., many sessions of long duration in a few weeks) or prolonged (i.e., a few hours of intervention delivered throughout many weeks). The present AR-TMI was effective in six children after six 15-min intervention sessions. Therefore, this is a short and effective targeted RJA intervention that is suitable for situations in which children’s or interventionists’ time is limited (e.g., therapy sessions after school).

- Availability and accessibility

Another advantage of this intervention study compared to previous TMIs (e.g., [64,65,66,67,68]) is the availability of the HW and SW that are needed for its implementation. The Pictogram Room only requires HW that is commercially available and relatively inexpensive when compared to other sophisticated and expensive technologies such as robots. Besides, most of the required HW (e.g., a PC, a projector) is often available at schools in the Western world. Furthermore, the SW is free to download in different languages making it accessible to a greater number of people. All the above-mentioned advantages suggest that the Pictogram Room might be an appropriate system to be included in schools as a tool for staff to teach JA skills. Moreover, this AR-TMI has been designed to be delivered independently by one interventionist (e.g., teacher) in one-to-one play sessions, not requiring further staff resources. This is an advantage compared to what has been found in previous TMI studies in which the researchers had to operate the technology to deliver prompts and respond according to children’s behaviours [65,66].

- Ecological validity

Literature has highlighted the importance of implementing JA interventions in autistic children which are delivered not only by therapists but also by teachers [57], parents [41,59], siblings [43] and peers [37] due to the need for more ecologically-valid interventions. For this purpose, in these aforementioned studies, the people who delivered the intervention were trained in specific teaching methods such as ABA for which they invested money, time and effort in order to gain the appropriate skills to deliver the intervention effectively.

An important advantage of the present intervention is that the Pictogram Room is cost-effective in the sense that it is an easy-to-learn system that can be used by anyone who wants to teach RJA skills without the need to attend long and sophisticated training programmes. This advantage also facilitates the implementation of the same intervention across people with different roles (e.g., teachers, clinicians, parents) and across settings (e.g., school, therapy centre, home) increasing the chances for the children to practise and generalise the learned RJA skills.

- Designing spectrum-comprehensive intervention studies

The findings have shown that this TMI has been beneficial for one autistic child with higher cognitive abilities and a lower level of support needed (i.e., P3; Leiter-R: 80; Level of autism support needed: (2) as well as for one autistic child with lower cognitive abilities and a higher level of support needed (i.e., P2; Leiter-R: 52; Level of autism support needed: (3). By having the potential to benefit children of a wide range of cognitive abilities and with a wide range of characteristics associated with being on the autism spectrum, the current TMI study is more inclusive than many others which tend to target autistic individuals with higher cognitive abilities and lower levels of support needed. This assumption has been based not only on the diagnostic information that was available in the archives of the autism unit but also on additional and updated information that was collected by the researcher (i.e., SCQ and Leiter-R).

It is a significant finding that five of the six participants mastered following the dummy’s gaze and pointing to the right poster or turtle. P1 and P2 mastered to point to the right poster but they did not master to point to the right turtle. Given that two of the same coloured turtles were used in half of the trials, this result cannot be attributed to the possibility that the children were making choices based on their preferences. Therefore, it is suspected that if they had been through a few more sessions, these two children would have mastered pointing to the right turtle as they mastered pointing to the right poster. The finding that P5 did not master pointing to the right poster or to the right turtle throughout the intervention should not be fully associated with his low cognitive abilities, since P2, who scored lower than P5 in Leiter-R, correctly pointed to the right poster in all the trials of two sessions. Still, P5’s improvements should be considered significant because his ADHD symptoms may have made interacting with an AR system even more difficult. He needed a relatively higher number of verbal and physical supports while playing games than the other five participants. Future studies could explore the impact that ADHD symptoms may have on children’s learning outcomes in an AR or VR-TMI.

- Engagement

All children were engaged in the Learning Phase and Intervention Phase sessions, most probably due to the attraction they felt towards the stimuli and personalised rewards provided by the Pictogram Room. This finding may contribute to the widespread belief that participant engagement is an important condition that leads to better learning outcomes for autistic individuals [47]. There are various reasons why the use of the Pictogram Room may have been so engaging for all six participants. Firstly, all the stimuli were presented visually with reinforcing audio outputs, and verbal language was reduced to a minimum. The consideration of these features is in line with previous studies and intervention methods highlighting the importance of using visual stimuli and minimising the use of complex verbal language with autistic individuals [99,100]. Secondly, the stimuli were personalised for each participant with their favourite pictures, videos and songs. This echoes other evidence-based intervention methods which indicate the relevance of building interventions on each child’s preferences and interests because this facilitates learning outcomes (e.g., PRT, ESDM). Lastly but importantly, each session with the Pictogram Room was set for a short duration (i.e., 15 min) as the TEACCH approach [88] and other sources on how to teach autistic individuals [101] recommend. However, there was no specific timing for completing the games so participants could play and learn at their own pace. Literature indicates that many autistic children might have gone through many unsuccessful experiences at school and might need more time to build self-confidence [102]. By giving them time to successfully complete an activity, frustration and other negative emotions may appear less often, increasing the engagement opportunities and promoting learning outcomes [103].

### 4.4. Limitations

One of the limitations of this study might be the use of the ESCS, a scale which was designed to be used with younger children (i.e., from 8 to 30 months of age) [93]. However, as it has been stated [31], the lack of measures of JA for older individuals—beyond measures used in imaging studies, and latency and eye-tracking measures—is a significant gap in the field of autism research. In the present study, this constraint has been addressed by developing ad hoc RJA skills assessments, although their reliability and validity properties could not be evaluated. This should motivate future research directed to the development of standardised JA assessment tools for older children. Additionally, it is worth mentioning that the psychometric properties of a JA protocol aimed at children between 2 and 12 years old have been initially examined [104] but more need to be done. Additionally, a protocol to systematically evaluate the procedural fidelity of the intervention implementation was not used in this study. However, the researcher was present in all of the study sessions ensuring the adequate application of the established procedures. This is a study carried out in a specific context with a relatively small sample. Conducting further studies in other contexts including more children would help to investigate the generalisation of the study outcomes as well as the evaluation of moderating variables such as the setting or the country.

### 4.5. Implications for Research and Practice

#### 4.5.1. Previous Experiences and Adaptations Using an AR System

The findings showed that some of the children needed specific guidance during the Learning Phase because, instead of keeping the initial distance from the IDW, they either walked forward to touch the screen or looked for the stimuli in the real world, walking out of the game zone. An explanation for the first response could be that the participants were behaving according to previous experiences they had using the same HW (i.e., an IDW) in school activities in which they had to touch the screen. Nevertheless, both responses recall the difficulties that autistic individuals have in processing the sense of agency [105] and forms of self-reflective awareness such as self-consciousness [106]. Considering that this has been the first experience of the participant children in using AR, it is not surprising that they needed support to learn and internalise the processes that are involved in the visual representation of the self [107] through a virtual skeleton. Learning to see themselves as agents of action reflected on a screen who are able to interact with stimuli that are not available in the real world but in a virtual world is fundamental for the effectiveness of an AR-TMI.

Based on these findings, future studies may consider: (a) using different HW that may not trigger responses based on previous experiences (e.g., a white wall instead of an IDW); (b) defining the game zone using visual cues (e.g., applying colourful tape on the floor) as recommended in the literature [88]; and (c) including as many trials as needed during the Learning Phase to ensure that all participants can successfully use AR before they start the Intervention Phase. The latter recommendation would also contribute to the abovementioned substantial need to give autistic children enough time to succeed in new activities and build their self-confidence. These two features could make the children feel more confident in using the AR system and might also have a direct impact on the effect of the intervention.

#### 4.5.2. The Meaning of Selecting Appropriate Materials

One of the most remarkable findings of this TMI study is the generalisation that was observed when the children correctly followed the researcher’s gaze and pointed to the target poster when this was placed behind them in the ESCS post-assessment; this response was not practised in the virtual world during the intervention. Thus, although literature highlights the difficulties for autistic people in transferring and generalising learned skills, this study demonstrated the ability of six children to follow a human’s gaze after having practised following the virtual child’s gaze. One factor that could explain this positive finding is related to the use of the dummy for assessing RJA after each intervention session. The dummy was similar to the virtual child in the sense that both had a non-expressive face that only moved the irises of their eyes. The dummy was also similar to the human in the sense that both were tangible and present in the real world. However, the three agents were different in the sense that the participants needed to process much less information with the virtual child and the dummy than with the human’s face, which often expresses complex information that is difficult to process at once for many autistic individuals [108]. Although the dummy was used as an evaluating tool, it might have become to some extent a facilitating tool for bridging the virtual and the real world. Future AR or VR-TMI studies could explore this thought.

#### 4.5.3. Using Other Pictogram Room Features and Taking Advantage of New Technology Developments

In this study, the *Gaze following* game was used to teach RJA skills to the participant children because this is the only one that provides gaze following training. However, the Pictogram Room includes other games (e.g., *Pointing at each other* of the *Pointing* set of video games; *Imitating the rhythm* of the *Imitation* set of video games) that can further contribute to the enhancement of not only RJA but also IJA, which is extremely important as IJA has been reported to have received less attention from a technology-based intervention perspective [109]. Future studies can explore the effects of a more comprehensive intervention that uses a combination of different games on the enhancement of JA and also other relevant related skills such as body knowledge or imitation.

On another note, it would be valuable to track the participant children’s gaze in the *Gaze following* game, and in the Which poster/turtle is the dummy looking at? and Which poster/turtle is s/he looking at? assessments to observe if they look at the irises of the virtual child, the dummy and the evaluator. This additional information can contribute to better knowing if the changes that are observed in the children’s responses are associated with changes in their visual fixation patterns. Considering the potential of cutting-edge eye-tracking systems that are arising in the field [110,111], future studies can use the Pictogram Room tool in combination with glasses or head-mounted devices to get a broader understanding of the intervention effects on the children’s behavioural responses.

At the time of writing this paper, software developers are working on a new version of Pictogram Room that will be launched in 2022 with updated games that will allow interaction with smart objects. It will also include an internal system of data capturing and will work with newer operating systems and cutting-edge sensors. All these new features will open valuable research opportunities such as the evaluation of the user’s body movements and behavioural responses to Pictogram Room stimuli.

#### 4.5.4. The Importance of the Setting and the Interventionists

Two important factors that have contributed to the success of this TMI study have been (a) that it has been conducted in a school, and (b) that the school staff was involved throughout the study. As advised in Reichow et al.’s [85] evaluative method, both these factors are principal for the social validity of intervention studies with autistic children. This study shows in many different ways why this is truly important. To start, the school is a familiar setting where children go to learn new skills. Moreover, teachers are highly motivated for contributing to this learning. This may be specifically the case for the participant teachers with theoretical expertise in special educational needs and many years of experience in teaching autistic children. All the information they shared with the researcher with regard to the participants’ communication system, abilities, special needs, sensory and time management difficulties, interests and preferences was of paramount importance for the success of the study. Besides, this study showed how school staff can be fully involved in the study and still be blind to some key points of it (e.g., in which number session each child was at a given time), efficiently protecting the study from internal validity threats.

Considering all these aspects, future studies may consider the relevance of conducting these types of interventions in natural environments such as schools. It will also be principal to rely on the participation of school staff who directly give support to the autistic children and, preferably, has extensive expertise in teaching them. As widely advised by relevant literature, the children’s sensory processing difficulties should be carefully considered [112,113]. A negative experience (e.g., too loud music) with the Pictogram Room or any other piece of technology could cause, apart from distress to the child, feelings of rejection towards it which would be hard to extinguish at a later stage. Finally, whenever possible, children’s communicative system (e.g., PECS communication book) should be available for them to say, request or respond anytime.

## 5. Conclusions

In the present study, the effects of an AR-TMI to enhance JA skills in autistic children were examined. The objective of the study was to improve the RJA skills of following another person’s gaze and pointing to an object of shared attention through the use of the Pictogram Room. A multiple baseline SSED was applied to six children who improved performance in RJA following the intervention. Improvements were maintained over time and generalised to real-world situations. These findings demonstrate that autistic children can improve their RJA skills with a targeted and engaging intervention. This has been the first study focusing on the use of the Pictogram Room to enhance RJA skills in autistic children. This AR system has been presented as a novel accessible and affordable easy-to-use technology that can be tried with individuals with a wide range of special needs obtaining significant improvements in a relatively short period. This study explored the impact of the Pictogram Room on JA but, given the variety of games that the system offers, it could be used for enhancing other skills such as body knowledge or imitation. Relevant implications for future research and practice have been addressed, the selection of a natural setting and a familiar interventionist, being the principal ones. Finally, in an effort to design and implement the study with high rigour and strength levels, Reichow et al.’s [85] evaluative method was significantly considered. As a result, this research report allows for replication studies [114] and it is suitable for being included in possible future meta-analytic reviews, contributing so to the promotion of evidence-based practices in the field.

## Figures and Tables

**Table 1:** Characteristics of the six participants in this study.

Participant’s Code	Sex	Chronological Age (Years, Months)	DSM-5 (Diagnosis)	GARS-2	SCQ	Leiter-R (IQ)
P1	Male	7, 10	Autism (Level 3) with intellectual and language impairment	111	15	63
P2	Female	8, 6	Autism (Level 3) with intellectual and language impairment	-	28	52
P3	Male	6, 7	Autism (Level 2) with intellectual and language impairment	81	13	80
P4	Male	5, 1	Autism (Level 3) with intellectual and language impairment	96	18	70
P5	Male	5, 5	Autism (Level 3) with intellectual and language impairment, with ADHD	98	31	54
P6	Male	3, 6	Autism (Level 3) with intellectual and language impairment	87	12	77

Note. The GARS-2 [86] score for P2 cannot be provided because it was not available in the archives of the school. ADHD: Attention Deficit Hyperactivity Disorder [87].

**Table 2:** Assessment tools used for describing participant characteristics and evaluating RJA skills.

Assessment Tool	Purpose	Forms/Items Used	Scores
The Social Communication Questionnaire (SCQ; [90])	To confirm the participants’ autism diagnoses and severity levels ^1^	The current form of this scale, which focuses on behaviours observed during the most recent three months of a child’s life.	≥15, highly suggestive of autism
The Leiter International Performance Scale, Revised [91]	To get the participants’ intelligence quotient (IQ) scores	The brief version of this non-verbal test, which consists of two main broad areas: Visualisation and Reasoning.	IQ < 85, suggests a below-average cognitive ability; IQ < 70, is highly suggestive of intellectual disability (ID)
The Autism Diagnostic Observation Schedule, Second Edition (ADOS-2; [92])	To measure the participants’ RJA skills	The RJA item of module 1 and the corresponding toy (i.e., remote-controlled toy animal).	Following the ADOS-2 scoring system for this item, scores ranged from 0 (the child used the orientation of the evaluator’s eyes as a cue to look towards the target, without the need for pointing) to 3 (the child did not orient to the object even when the object was activated)
The Early Social Communication Scales (ESCS; [93])	To measure the participants’ RJA skills	The RJA module includes an assessment in which the child sits in a chair in front of the evaluator. Four posters are placed on four different points related to the child’s position: left or right (L/R RJA), and back left or back right (Behind RJA). The evaluator looks at one of the posters and the child should respond by turning his/her head and looking in the same direction as the evaluator. In this study, six trials for L/R RJA and six trials for Behind RJA were performed with each child during each assessment session.	The coefficient between the number of trials in which the child presented RJA and the total number of trials

Note. ^1^ The participants’ autism diagnoses and severity levels were provided by external clinicians and obtained by the researcher from the archives of the autism unit before the start of the study. The participants’ autism diagnoses were based on the *Diagnostic and Statistical Manual of Mental Disorders, Fifth Edition* (DSM-5; [87]) and the severity levels had been obtained with The Gilliam Autism Rating Scale, Second Edition (GARS-2; [86]). This consists of 42 items describing the (a) stereotyped behaviour, (b) communication and (c) social interaction of a person on the autism spectrum. A total score of 70 or higher indicates that the person possibly is autistic and a total score of 85 or higher indicates that the person is very likely to be autistic.

**Table 3:** Description of the phases of the study.

Phases of the Study	Groups (1–3)	Weeks (1–12)	Sessions (1–9)	Assessments	Pictogram Room Games
Pre-baseline Phase					
One-off assessments	1–3	1	1	SCQ, Leiter-R	
Pre-assessments	1–3	1	1	ADOS-2, ESCS, Which poster is s/he looking at? Which turtle is s/he looking at?	
Baseline Phase	1	2	3	Which poster is the dummy looking at? Which turtle is the dummy looking at?	
	2	2–3	6		
	3	2–4	9		
Learning Phase	1	3	3	Which poster is the dummy looking at? Which turtle is the dummy looking at?	Touch
	2	4	3		
	3	5	3		
Intervention Phase	1	4–5	6	Which poster is the dummy looking at? Which turtle is the dummy looking at?	Gaze following
	2	5–6	6		
	3	6–7	6		
Post-intervention Phase					
Post-assessments	1–3	8	1	ADOS-2, ESCS, Which poster is s/he looking at? Which turtle is s/he looking at?	
Follow-up assessments	1–3	12	2	Which poster is the dummy looking at? Which turtle is the dummy looking at? Which poster is s/he looking at? Which turtle is s/he looking at?	

**Table 4:** Operationalisation of measured variables.

Variable	Assessment Tool	Assessment Unit	Scoring System	Scoring System Description
v1	Pictogram Room games	General performance	0–3	0 = player did not complete the level game 3 = player completed the level game on his/her own
v2	Which poster is the dummy looking at?	Percentage of correct responses	0–100%	0% = 0 correct responses in 10 trials 100% = 10 correct responses in 10 trials
v3	Which turtle is the dummy looking at?	Percentage of correct responses	0–100%	0% = 0 correct responses in 10 trials 100% = 10 correct responses in 10 trials
v4	Which poster is s/he looking at?	The proportion of correct responses	0–1	0 = 0 correct responses in 10 trials 1 = 10 correct responses in 10 trials
v5	Which turtle is s/he looking at?	The proportion of correct responses	0–1	0 = 0 correct responses in 10 trials 1 = 10 correct responses in 10 trials
v6	ESCS	L/R RJA Behind RJA	0–1	0 = 0 correct responses in 6 trials 1 = 6 correct responses in 6 trials
v7	ADOS-2	RJA	0–3	0 = the lowest RJA level 3 = the highest RJA level

**Table 5:** Pre, post and follow-up measures, IOA and Kappa scores for v4–7.

	Participant	V4			V5			V6: ESCS				V7: ADOS-2	
		Poster			Turtle			L/R RJA		Behind RJA			
		Pre	Post	F-Up	Pre	Post	F-Up	Pre	Post	Pre	Post	Pre	Post
Group 1	P1	0.30	00.90	0.90	0.30	1	1	0.17	0.83	0	0.67	2	2
	P2	0.30	1	1	0.20	1	1	0.33	0.83	0.17	0.67	2	1
Group 2	P3	0.30	1	1	0.30	1	1	0.67	1	0.50	1	1	0
	P4	0.20	1	0.90	0.30	0.90	0.90	0.50	0.83	0.17	0.83	2	2
Group 3	P5	0.40	0.80	0.90	0.20	0.80	0.70	0.17	0.67	0	0.33	3	3
	P6	0.30	1	1	0.20	1	1	0.33	1	0.33	0.83	2	1
IOA		1			1			0.97				0.92	
Kappa		1			1			0.94				0.88	

**Table 6:** Research report rigour rating.

Rigour Rating											
Primary Quality Indicators						Secondary Quality Indicators					
PART	IV	DV	BSLN	VIS AN	EXP CON	IOA	KAP	FID	BR	G/M	SV
H	H	H	H	H	H	Y	Y	N	Y	Y	Y

Note. PART: participant characteristics; IV: independent variable; DV: dependent variable; BSLN: baseline condition; VIS AN: visual analysis; EXP CON: experimental control; IOA: interobserver agreement; KAP: Kappa; FID: fidelity; BR: blind raters; G/M: generalisation and/or maintenance; SV: social validity; H: high quality; A: acceptable quality; U: unacceptable quality; Y: there is evidence; N: there is no evidence. Rating form adapted from [98] (p. 38).

## Data Availability

G.H. holds the copyright of Figure 1 and Figure 3, which are available at the Pictogram Room’s website: http://www.pictogramas.org/proom (accessed on 13 January 2022). P.P.-F. holds the copyright of the other figures and all of the tables that have been reproduced from her PhD thesis, which is openly available at https://roderic.uv.es/bitstream/handle/10550/58695/Pérez-Fuster_Patricia_Doctoral%20Thesis.pdf?sequence=1 (accessed on 13 January 2022). The data presented in this study are openly available in Open Science Framework. DOI 10.17605/OSF.IO/ZEV5A. In detail, all the information concerning the research study’s data can be found here: https://mfr.osf.io/render?url=https://osf.io/bmne4/?direct%26mode=render%26action=download%26mode=render (accessed on 13 January 2022).

## References

[1] Charman T., Swettenham J., Baron-Cohen S., Cox A., Baird G., Drew A. (1997). Infants with autism: An investigation of empathy, pretend play, joint attention, and imitation. Dev. Psychol..

[2] Charman T., Swettenham J., Baron-Cohen S., Cox A., Baird G., Drew A. (1998). An experimental investigation of social-cognitive abilities in infants with autism: Clinical implications. Infant Ment. Health J..

[3] Mundy P., Gomes A. (1998). Individual Differences in Joint Attention Skill Development in the Second Year. Infant Behav. Dev. Int. Interdiscip. J..

[4] Mundy P., Newell L. (2007). Attention, Joint Attention, and Social Cognition. Curr. Dir. Psychol. Sci..

[5] Franco F., Butterworth G. (1996). Pointing and Social Awareness: Declaring and Requesting in the Second Year. J. Child Lang..

[6] Mundy P., Crowson M. (1997). Joint Attention and Early Social Communication: Implications for Research on Intervention with Autism. J. Autism Dev. Disord..

[7] Brooks R., Meltzoff A.N. (2005). The Development of Gaze Following and Its Relation to Language. Dev. Sci..

[8] Leekam S.R., Hunnisett E., Moore C. (1998). Targets and cues: Gaze-following in children with autism. J. Child Psychol. Psychiatry.

[9] Sigman M., Ruskin E. (1999). Continuity and Change in the Social Competence of Children with Autism, Down Syndrome, and Developmental Delays. Monogr. Soc. Res. Child Dev..

[10] Frith U., Frith C. (2001). The biological basis of social interaction. Curr. Dir. Psychol. Sci..

[11] Meltzoff A.N., Brooks R., Malle B.F., Moses L.J., Baldwin D.A. (2001). “Like me” as a building block for understanding other minds: Bodily acts, attention, and intention. Intentions and Intentionality: Foundations of Social Cognition.

[12] Tomasello M., Dunham P.J., Moore C. (1995). Joint attention as social cognition. Joint Attention: Its Origins and Role in Development.

[13] Baron-Cohen S. (1995). Mindblindness: An Essay on Autism and Theory of Mind.

[14] Charman T., Baron-Cohen S., Swettenham J., Baird G., Cox A., Drew A. (2000). Testing joint attention, imitation, and play as infancy precursors to language and theory of mind. Cogn. Dev..

[15] Bakeman R., Adamson L.B. (1984). Coordinating Attention to People and Objects in Mother-Infant and Peer-Infant Interaction. Child Dev..

[16] Butterworth G., Jarrett N. (1991). What minds have in common is space: Spatial mechanisms serving joint visual attention in infancy. Br. J. Dev. Psychol..

[17] Moore C., Corkum V. (1998). Infant gaze following based on eye direction. Br. J. Dev. Psychol..

[18] Morissette P., Ricard M., Décarie T.G. (1995). Joint visual attention and pointing in infancy: A longitudinal study of comprehension. Br. J. Dev. Psychol..

[19] Mundy P., Sigman M.D., Ungerer J., Sherman T. (1986). Defining the social deficits of autism: The contribution of non-verbal communication measures. Child Psychol. Psychiatry Allied Discip..

[20] Mundy P., Card J., Fox N. (2000). EEG correlates of the development of infant joint attention skills. Dev. Psychobiol..

[21] Gillespie-Lynch K., Elias R., Escudero P., Hutman T., Johnson S.P. (2013). Atypical gaze following in autism: A comparison of three potential mechanisms. J. Autism Dev. Disord..

[22] Mundy P., Sigman M., Kasari C. (1994). Joint attention, developmental level, and symptom presentation in autism. Dev. Psychopathol..

[23] Nation K., Penny S. (2008). Sensitivity to eye gaze in autism: Is it normal? Is it automatic? Is it social?. Dev. Psychopathol..

[24] Carpenter M., Pennington B.E., Rogers S.J. (2002). Interrelations among social-cognitive skills in young children with autism. J Autism Dev. Disord..

[25] Dawson G., Meltzoff A.N., Osterling J., Rinaldi J., Brown E. (1998). Children with autism fail to orient to naturally occurring social stimuli. J. Autism Dev. Disord..

[26] Paparella T., Goods K.S., Freeman S., Kasari C. (2011). The emergence of nonverbal joint attention and requesting skills in young children with autism. J. Commun. Disord..

[27] Charman T., Frith U., Hill E. (2003). Why is joint attention a pivotal skill in autism?. Autism: Mind and Brain.

[28] Dawson G., Toth K., Abbott R., Osterling J., Munson J., Estes A., Liaw J. (2004). Early Social Attention Impairments in Autism: Social Orienting, Joint Attention, and Attention to Distress. Dev. Psychol..

[29] Morales M., Mundy P., Delgado C.E.F., Yale M., Messinger D., Neal R., Schwartz H.K. (2000). Responding to Joint Attention across the 6- through 24-Month Age Period and Early Language Acquisition. J. Appl. Dev. Psychol..

[30] Gangi D.N., Ibañez L.V., Messinger D.S. (2014). Joint attention initiation with and without positive affect: Risk group differences and associations with ASD symptoms. J. Autism Dev. Disord..

[31] Mundy P.C. (2016). Autism and Joint Attention: Development, Neuroscience, and Clinical Fundamentals.

[32] Whalen C., Schreibman L., Ingersoll B. (2006). The Collateral Effects of Joint Attention Training on Social Initiations, Positive Affect, Imitation, and Spontaneous Speech for Young Children with Autism. J. Autism Dev. Disord..

[33] Isaksen J., Holth P. (2009). An operant approach to teaching joint attention skills to children with autism. Behav. Interv..

[34] Martins M.P., Harris S.L. (2006). Teaching Children with Autism to Respond to Joint Attention Initiations. Child Fam. Behav. Ther..

[35] Taylor B.A., Hoch H. (2008). Teaching children with autism to respond to and initiate bids for joint attention. J. Appl. Behav. Anal..

[36] Wong C.S., Kasari C., Freeman S., Paparella T. (2007). The acquisition and generalization of joint attention and symbolic play skills in young children with autism. Res. Pract. Pers. Sev. Disabil..

[37] Zercher C., Hunt P., Schuler A., Webster J. (2001). Increasing Joint Attention, Play and Language through Peer Supported Play. Autism.

[38] Jones E.A., Carr E.G., Feeley K.M. (2006). Multiple Effects of Joint Attention Intervention for Children with Autism. Behav. Modif..

[39] Whalen C., Schreibman L. (2003). Joint attention training for children with autism using behavior modification procedures. J. Child Psychol. Psychiatry.

[40] Jones E.A. (2009). Establishing response and stimulus classes for initiating joint attention in children with Autism. Res. Autism Spectr. Disord..

[41] Rocha M.L., Schreibman L., Stahmer A.C. (2007). Effectiveness of training parents to teach joint attention in children with autism. J. Early Interv..

[42] Vismara L.A., Lyons G.L. (2007). Using perseverative interests to elicit joint attention behaviors in young children with autism: Theoretical and clinical implications for understanding motivation. J. Posit. Behav. Interv..

[43] Ferraioli S.J., Harris S.L. (2011). Teaching Joint Attention to Children with Autism Through A Sibling-Mediated Behavioral Intervention. Behav. Interv..

[44] Pierce K., Schreibman L. (1995). Increasing complex social behaviors in children with autism: Effects of peer-implemented pivotal response training. J. Appl. Behav. Anal..

[45] Ingersoll B., Schreibman L. (2006). Teaching Reciprocal Imitation Skills to Young Children with Autism Using a Naturalistic Behavioral Approach: Effects on Language, Pretend Play, and Joint Attention. J. Autism Dev. Disord..

[46] Ingersoll B. (2012). Effect of a focused imitation intervention on social functioning in children with autism. J. Autism Dev. Disord..

[47] Rogers S.J., Dawson G. (2010). Early Start Denver Model for Young Children with Autism: Promoting Language, Learning, and Engagement.

[48] Rogers S.J., Hayden D., Hepburn S., Charlifue-Smith R., Hall T., Hayes A. (2006). Teaching young nonverbal children with autism useful speech: A pilot study of the Denver model and PROMPT interventions. J. Autism Dev. Disord..

[49] Vismara L.A., Rogers S.J. (2008). The Early Start Denver model: A case study of an innovative practice. J. Early Interv..

[50] Vismara L.A., Colombi C., Rogers S.J. (2009). Can one hour per week of therapy lead to lasting changes in young children with autism?. Autism.

[51] Schertz H.H., Odom S.L. (2007). Promoting joint attention in toddlers with autism: A parent-mediated developmental model. J. Autism Dev. Disord..

[52] Schertz H.H., Odom S.L., Baggett K.M., Sideris J.H. (2013). Effects of Joint Attention Mediated Learning for toddlers with autism spectrum disorders: An initial randomized controlled study. Early Child. Res. Q..

[53] Kasari C., Gulsrud A.C., Shire S.Y., Strawbridge C. (2021). The JASPER Model for Children with Autism: Promoting Joint Attention, Symbolic Play, Engagement, and Regulation.

[54] Gulsrud A.C., Hellemann G.S., Freeman S.F.N., Kasari C. (2014). Two to ten years: Developmental trajectories of joint attention in children with ASD who received targeted social communication interventions. Autism Res..

[55] Kasari C., Freeman S., Paparella T. (2006). Joint attention and symbolic play in young children with autism: A randomized controlled intervention study. J. Child Psychol. Psychiatry Allied Discip..

[56] Kasari C., Gulsrud A., Freeman S., Paparella T., Hellemann G. (2012). Longitudinal follow-up of children with autism receiving targeted interventions on joint attention and play. J. Am. Acad. Child Adolesc. Psychiatry.

[57] Lawton K., Kasari C. (2012). Teacher-implemented joint attention intervention: Pilot randomized controlled study for preschoolers with autism. J. Consult. Clin. Psychol..

[58] Kasari C., Gulsrud A., Paparella T., Hellemann G., Berry K. (2015). Randomized comparative efficacy study of parent-mediated interventions for toddlers with autism. J. Consult. Clin. Psychol..

[59] Kasari C., Gulsrud A.C., Wong C., Kwon S., Locke J. (2010). Randomized Controlled Caregiver Mediated Joint Engagement Intervention for Toddlers with Autism. J. Autism Dev. Disord..

[60] Kasari C., Lawton K., Shih W., Barker T.V., Landa R., Lord C., Orlich F., King B., Wetherby A., Senturk D. (2014). Caregiver-mediated intervention for low-resourced preschoolers with autism: An RCT. Pediatrics.

[61] Kasari C., Paparella T., Freeman S., Jahromi L.B. (2008). Language outcome in autism: Randomized comparison of joint attention and play interventions. J. Consult. Clin. Psychol..

[62] Kasari C., Kaiser A., Goods K., Nietfeld J., Mathy P., Landa R., Murphy S., Almirall D. (2014). Communication interventions for minimally verbal children with autism: A sequential multiple assignment randomized trial. J. Am. Acad. Child Adolesc. Psychiatry.

[63] Shih W., Shire S., Chang Y., Kasari C. (2021). Joint engagement is a potential mechanism leading to increased initiations of joint attention and downstream effects on language: JASPER early intervention for children with ASD. J. Child Psychol. Psychiatry.

[64] Cheng Y., Huang R. (2012). Using virtual reality environment to improve joint attention associated with pervasive developmental disorder. Res. Dev. Disabil..

[65] Goodrich M.A., Colton M., Brinton B., Fujiki M., Atherton J.A., Robinson L., Ricks D., Maxfield M.H., Acerson A. (2012). Incorporating a Robot into an Autism Therapy Team. IEEE Intell. Syst..

[66] Costa S., Lehmann H., Dautenhahn K., Robins B., Soares F. (2015). Using a Humanoid Robot to Elicit Body Awareness and Appropriate Physical Interaction in Children with Autism. Int. J. Soc. Robot..

[67] Tapus A., Peca A., Aly A., Pop C., Jisa L., Pintea S., Rusu A.S., David D.O. (2012). Children with autism social engagement in interaction with Nao, an imitative robot: A series of single case experiments. Interact. Stud. Soc. Behav. Commun. Biol. Artif. Syst..

[68] Warren Z.E., Zheng Z., Swanson A.R., Bekele E., Zhang L., Crittendon J.A., Weitlauf A.F., Sarkar N. (2015). Can Robotic Interaction Improve Joint Attention Skills?. J. Autism Dev. Disord..

[69] Sani-Bozkurt S., Bozkus-Genc G. (2021). Social Robots for Joint Attention Development in Autism Spectrum Disorder: A Systematic Review. Int. J. Disabil. Dev. Educ..

[70] Lee K. (2012). Augmented Reality in Education and Training. TechTrends.

[71] Herrera G., Jordan R., Vera L. (2006). Agency and presence: A common dependence on subjectivity?. Presence-Teleoper. Virtual Environ..

[72] Parsons S. (2016). Authenticity in Virtual Reality for assessment and intervention in autism: A conceptual review. Educ. Res. Rev..

[73] Herrera G., Jordan R., Gimeno J. Exploring the advantages of augmented reality for intervention in asd. Proceedings of the 2nd World Autism Congress & Exhibition, Autism Safari.

[74] Kientz J.A., Hayes G.R., Goodwin M.S., Gelsomini M., Abowd G.D. (2020). Interactive Technologies and Autism.

[75] Bai Z., Blackwell A.F., Coulouris G. (2015). Using Augmented Reality to Elicit Pretend Play for Children with Autism. IEEE Trans. Vis. Comput. Graph..

[76] Bhattacharya A., Gelsomini M., Pérez-Fuster P., Abowd G.D., Rozga A. (2015). Designing motion-based activities to engage students with autism in classroom settings. Proceedings of the 14th International Conference on Interaction Design and Children.

[77] Escobedo L., Nguyen D.H., Boyd L., Hirano S., Rangel A., Garcia-Rosas D., Tentori M., Hayes G. (2012). MOSOCO: A mobile assistive tool to support children with autism practicing social skills in real-life situations. Proceedings of the SIGCHI Conference on Human Factors in Computing Systems.

[78] Escobedo L., Tentori M., Quintana E., Favela J., Garcia-Rosas D. (2014). Using Augmented Reality to Help Children with Autism Stay Focused. MPRV.

[79] Liu R., Salisbury J.P., Vahabzadeh A., Sahin N.T. (2017). Feasibility of an Autism-Focused Augmented Reality Smartglasses System for Social Communication and Behavioral Coaching. Front. Pediatrics.

[80] Marto A., Almeida H.A., Gonçalves A. (2019). Using Augmented Reality in Patients with Autism: A Systematic Review. VipIMAGE 2019.

[81] Casas X., Herrera G., Coma I., Fernández M. (2012). A Kinect-based Augmented Reality System for Individuals with Autism Spectrum Disorders. Proceedings of the International Conference on Computer Graphics Theory and Applications (GRAPP).

[82] Herrera G., Casas X., Sevilla J., Rosa L., Pardo C., Plaza J., Jordan R., Le Groux S. (2012). Pictogram Room: Natural Interaction Technologies to Aid in the Development of Children with Autism. Annu. Clin. Health Psychol..

[83] Nadel J., Poli G. (2018). Evaluating and training body knowledge in autism via Kinect and Pictogram Room. Enfance.

[84] Mademtzi M. (2016). The use of a Kinect-based technology within the school environment to enhance sensory-motor skills of children with autism. Ph.D. Thesis.

[85] Reichow B., Volkmar F.R., Cicchetti D.V. (2008). Development of the Evaluative Method for Evaluating and Determining Evidence-Based Practices in Autism. J. Autism Dev. Disord..

[86] Gilliam J.E. (2006). Gilliam Autism Rating Scale.

[87] American Psychiatric Association (2013). Diagnostic and Statistical Manual of Mental Disorders.

[88] Mesibov G.B., Shea V., Schopler E. (2005). The TEACCH Approach to Autism Spectrum Disorders.

[89] Bondy A.S., Frost L.A. (1994). The picture exchange communication system. Focus Autistic Behav..

[90] Rutter M., Bailey A., Lord C. (2003). The Social Communication Questionnaire (SCQ).

[91] Roid G.H., Miller L.J. (1997). Leiter International Performance Scale, Revised (Leiter-R).

[92] Lord C., Rutter M., DiLavore P.C., Risi S., Gotham K., Bishop S.L. (2012). Autism Diagnostic Observation Schedule.

[93] Mundy P., Delgado C., Block J., Venezia M., Hogan A., Seibert J. (2003). Early Social Communication Scales (ESCS).

[94] Kazdin A.E. (1982). Single-case experimental designs in clinical research and practice. New Dir. Methodol. Soc. Behav. Sci..

[95] Leppink J. (2022). Bridging research and practice in health professions education: Single case designs. Asia Pac. Sch..

[96] Cohen J. (1960). A coefficient of agreement for nominal scales. Educ. Psychol. Meas..

[97] Parker R.I., Hagan-Burke S., Vannest K. (2007). Percentage of All Non-Overlapping Data (PAND): An Alternative to PND. J. Spec. Educ..

[98] Reichow B., Reichow B., Doehring P., Cicchetti D.V., Volkmar F.R. (2011). Development, Procedures, and Application of the Evaluative Method for Determining Evidence-Based Practices in Autism. Evidence-Based Practices and Treatments for Children with Autism.

[99] Hume K., Odom S. (2007). Effects of an Individual Work System on the Independent Functioning of Students with Autism. J. Autism Dev. Disord..

[100] Kossyvaki L., Jones G., Guldberg K. (2012). The effect of adult interactive style on the spontaneous communication of young children with autism at school. Br. J. Spec. Educ..

[101] Jordan R., Powell S. (1995). Understanding and Teaching Children with Autism.

[102] Jackson L. (2002). Freaks, Geeks and Asperger Syndrome: A User Guide to Adolescence.

[103] Jordan R. (1999). Autistic Spectrum Disorders: An Introductory Handbook for Practitioners.

[104] Nowell S.W., Watson L.R., Faldowski R.A., Baranek G.T. (2018). An initial psychometric evaluation of the joint attention protocol. J. Autism Dev. Disord..

[105] Russell J. (1996). Agency: Its Role in Mental Development.

[106] Hobson R.P. (1993). Autism and the Development of Mind.

[107] Newbutt N., Felicia P. (2014). Representations of the self in classroom virtual worlds: An autism perspective. Game-Based Learning: Challenges and Opportunities.

[108] Dawson G., Webb S.J., McPartland J. (2005). Understanding the Nature of Face Processing Impairment in Autism: Insights from Behavioral and Electrophysiological Studies. Dev. Neuropsychol..

[109] Nie G., Ullal A., Zheng Z., Swanson A.R., Weitlauf A.S., Warren Z.E., Sarkar N. (2021). An Immersive Computer-Mediated Caregiver-Child Interaction System for Young Children with Autism Spectrum Disorder. TNSRE.

[110] Chong E., Chanda K., Ye Z., Southerland A., Ruiz N., Jones R.M., Rozga A., Rehg J.M. (2017). Detecting Gaze Towards Eyes in Natural Social Interactions and Its Use in Child Assessment. Proceedings of the ACM on Interactive, Mobile, Wearable and Ubiquitous Technologies.

[111] Ambrose D., MacKenzie D.E., Ghanouni P., Neyedli H.F. (2021). Investigating joint attention in a guided interaction between a child with ASD and therapists: A pilot eye-tracking study. Br. J. Occup. Ther..

[112] Bogdashina O. (2003). Sensory Perceptual Issues in Autism and Asperger Syndrome: Different Sensory Experiences—Different Perceptual Worlds.

[113] Schaaf R.C., Toth-Cohen S., Johnson S.L., Outten G., Benevides T.W. (2011). The everyday routines of families of children with autism: Examining the impact of sensory processing difficulties on the family. Autism.

[114] Leppink J., Pérez-Fuster P. (2016). What is science without replication?. Perspect. Med. Educ..

[115] (2013). World Medical Association Ethical Principles for Medical Research Involving Human Subjects. Clin. Rev. Educ..

